# A prospective, open-label, multicenter, observational, postmarket study of the use of a 15 mg/mL hyaluronic acid dermal filler in the lips

**DOI:** 10.1111/jocd.12085

**Published:** 2014-06-09

**Authors:** Wolfgang G Philipp-Dormston, Said Hilton, Myooran Nathan

**Affiliations:** 1 Cologne Dermatology, Hautzentrum KölnCologne, Germany; 2 Medical Skin Center Dr HiltonDüsseldorf, Germany; 3 Allergan LtdParkway, Marlow, Bucks, UK

**Keywords:** dermal filler, hyaluronic acid, Juvéderm, lips, VOLBELLA, VYCROSS

## Abstract

Hyaluronic acid (HA)-based injectable fillers three-dimensionally restore the natural contours of the lips and perioral area, thereby reducing some signs of aging lips. To evaluate the short-term aesthetic impact of treatment with the HA dermal filler Juvéderm® VOLBELLA® with Lidocaine, formulated utilizing VYCROSS™ technology, for enhancement or correction of asymmetry of the lips, evaluated using a patient-centric approach. Sixty-two subjects were enrolled in this study, conducted at two sites in Germany. Primary endpoints were satisfaction with improvement, look and feel of the lips, assessed by subject and physician at first visit and 4 weeks post-treatment. Immediately after injection at first visit, 83.6% of subjects were *Extremely Satisfied*, *Very Satisfied* or *Satisfied* with improvement in the lips, which increased to 94.1% and 93.0% of subjects with/without top-up treatment at follow-up, respectively. After injection at first visit, 61.7% of subjects rated the look and feel of their lips as *Extremely Natural* or *Very Natural*, which increased to 75.0% and 93.0% of subjects with/without top-up treatment, respectively. The HA dermal filler was associated with minimal discomfort, bruising or swelling of the lips; almost two-thirds of subjects (62%) returned to social engagements on the same day. The high degree of subject satisfaction with aesthetic improvement in the lips, as well as the natural look and feel, indicates that this HA dermal filler represents an effective treatment option for patients requiring lip enhancement.

## Introduction

### Background

As a key aesthetic feature of the face, fullness and definition of the lips are associated with attractiveness, sensuality and youth. Similar to the skin, however, the lips are vulnerable to intrinsic and extrinsic factors that can change their appearance over time.^1^

These morphological changes of the lips become an obvious sign of aging. Repetitive and underlying action of the orbicularis oris muscle leads to the formation of visible fine vertical rhytides surrounding the lips. The effects of gravity coupled with loss of lip volume and support cause the upper lip to lengthen and the lip to fall vertically. As collagen production diminishes, the Cupid's bow and vermilion border lose their distinction,^1^ and the inability to conceal these signs of aging may be correlated with increased anxiety and depression.^2^

Treatment with dermal fillers can enhance the lips and perioral area, thereby reducing some of the signs of aging, and lip augmentation is currently recognized as one of the most common uses for dermal fillers.^3^–^6^ One of the most widely used filler substances on the market at present is hyaluronic acid (HA), which is a glycosaminoglycan-based polymer that is naturally produced by the body,^7^ and for more than 15 years these polymers have been cross-linked to extend the longevity of commercial HA products.^8^

A 15 mg/mL HA dermal filler has been developed using the VYCROSS™ technology platform (developed by Allergan Inc., Irvine, CA, USA) and is formulated using a majority of low molecular weight HA together with a minority of high molecular weight HA (>1 MDa). This formulation has more efficient cross-linking, which affects the rheology of the product in tissues and the hydrophilic properties of the HA gel. The optimized homogenous matrix is smooth rather than granular; this forms a highly malleable gel that is expected to distribute evenly in the treated tissue. These attributes result in a versatile product that can be used not only for lip enhancement, but also to treat fine lines. Finally, the inclusion of mainly low molecular weight HA in the gel, and a lower overall amount of HA, reduces the attraction of water from surrounding tissue, thus reducing the swelling of the gel (Data on File, RE1301025 Internal Report, Allergan Inc, 2012, unpublished data). This HA dermal filler also contains a small amount of non-cross-linked HA, which enhances the delivery of the gel to human tissues and gives the product a low extrusion force (Data on File, RE1210036 Internal Report, Allergan Inc, 2012).

A 15 mg/mL HA dermal filler has been developed using the VYCROSS™ technology platform (developed by Allergan Inc., Irvine, CA, USA) and is formulated using a majority of low molecular weight HA together with a minority of high molecular weight HA (>1 MDa). This formulation has more efficient cross-linking, which affects the rheology of the product in tissues and the hydrophilic properties of the HA gel. The optimized homogenous matrix is smooth rather than granular; this forms a highly malleable gel that is expected to distribute evenly in the treated tissue. These attributes result in a versatile product that can be used not only for lip enhancement, but also to treat fine lines. Finally, the inclusion of mainly low molecular weight HA in the gel, and a lower overall amount of HA, reduces the attraction of water from surrounding tissue, thus reducing the swelling of the gel (Data on File, RE1301025 Internal Report, Allergan Inc, 2012, unpublished data). This HA dermal filler also contains a small amount of non-cross-linked HA, which enhances the delivery of the gel to human tissues and gives the product a low extrusion force (Data on File, RE1210036 Internal Report, Allergan Inc, 2012).

A 15 mg/mL HA dermal filler has been developed using the VYCROSS™ technology platform (developed by Allergan Inc., Irvine, CA, USA) and is formulated using a majority of low molecular weight HA together with a minority of high molecular weight HA (>1 MDa). This formulation has more efficient cross-linking, which affects the rheology of the product in tissues and the hydrophilic properties of the HA gel. The optimized homogenous matrix is smooth rather than granular; this forms a highly malleable gel that is expected to distribute evenly in the treated tissue. These attributes result in a versatile product that can be used not only for lip enhancement, but also to treat fine lines. Finally, the inclusion of mainly low molecular weight HA in the gel, and a lower overall amount of HA, reduces the attraction of water from surrounding tissue, thus reducing the swelling of the gel (Data on File, RE1301025 Internal Report, Allergan Inc, 2012, unpublished data). This HA dermal filler also contains a small amount of non-cross-linked HA, which enhances the delivery of the gel to human tissues and gives the product a low extrusion force (Data on File, RE1210036 Internal Report, Allergan Inc, 2012).

A 15 mg/mL HA dermal filler has been developed using the VYCROSS™ technology platform (developed by Allergan Inc., Irvine, CA, USA) and is formulated using a majority of low molecular weight HA together with a minority of high molecular weight HA (>1 MDa). This formulation has more efficient cross-linking, which affects the rheology of the product in tissues and the hydrophilic properties of the HA gel. The optimized homogenous matrix is smooth rather than granular; this forms a highly malleable gel that is expected to distribute evenly in the treated tissue. These attributes result in a versatile product that can be used not only for lip enhancement, but also to treat fine lines. Finally, the inclusion of mainly low molecular weight HA in the gel, and a lower overall amount of HA, reduces the attraction of water from surrounding tissue, thus reducing the swelling of the gel (Data on File, RE1301025 Internal Report, Allergan Inc, 2012, unpublished data). This HA dermal filler also contains a small amount of non-cross-linked HA, which enhances the delivery of the gel to human tissues and gives the product a low extrusion force (Data on File, RE1210036 Internal Report, Allergan Inc, 2012).

A 15 mg/mL HA dermal filler has been developed using the VYCROSS™ technology platform (developed by Allergan Inc., Irvine, CA, USA) and is formulated using a majority of low molecular weight HA together with a minority of high molecular weight HA (>1 MDa). This formulation has more efficient cross-linking, which affects the rheology of the product in tissues and the hydrophilic properties of the HA gel. The optimized homogenous matrix is smooth rather than granular; this forms a highly malleable gel that is expected to distribute evenly in the treated tissue. These attributes result in a versatile product that can be used not only for lip enhancement, but also to treat fine lines. Finally, the inclusion of mainly low molecular weight HA in the gel, and a lower overall amount of HA, reduces the attraction of water from surrounding tissue, thus reducing the swelling of the gel (Data on File, RE1301025 Internal Report, Allergan Inc, 2012, unpublished data). This HA dermal filler also contains a small amount of non-cross-linked HA, which enhances the delivery of the gel to human tissues and gives the product a low extrusion force (Data on File, RE1210036 Internal Report, Allergan Inc, 2012).

A 15 mg/mL HA dermal filler has been developed using the VYCROSS™ technology platform (developed by Allergan Inc., Irvine, CA, USA) and is formulated using a majority of low molecular weight HA together with a minority of high molecular weight HA (>1 MDa). This formulation has more efficient cross-linking, which affects the rheology of the product in tissues and the hydrophilic properties of the HA gel. The optimized homogenous matrix is smooth rather than granular; this forms a highly malleable gel that is expected to distribute evenly in the treated tissue. These attributes result in a versatile product that can be used not only for lip enhancement, but also to treat fine lines. Finally, the inclusion of mainly low molecular weight HA in the gel, and a lower overall amount of HA, reduces the attraction of water from surrounding tissue, thus reducing the swelling of the gel (Data on File, RE1301025 Internal Report, Allergan Inc, 2012, unpublished data). This HA dermal filler also contains a small amount of non-cross-linked HA, which enhances the delivery of the gel to human tissues and gives the product a low extrusion force (Data on File, RE1210036 Internal Report, Allergan Inc, 2012).

A 15 mg/mL HA dermal filler has been developed using the VYCROSS™ technology platform (developed by Allergan Inc., Irvine, CA, USA) and is formulated using a majority of low molecular weight HA together with a minority of high molecular weight HA (>1 MDa). This formulation has more efficient cross-linking, which affects the rheology of the product in tissues and the hydrophilic properties of the HA gel. The optimized homogenous matrix is smooth rather than granular; this forms a highly malleable gel that is expected to distribute evenly in the treated tissue. These attributes result in a versatile product that can be used not only for lip enhancement, but also to treat fine lines. Finally, the inclusion of mainly low molecular weight HA in the gel, and a lower overall amount of HA, reduces the attraction of water from surrounding tissue, thus reducing the swelling of the gel (Data on File, RE1301025 Internal Report, Allergan Inc, 2012, unpublished data). This HA dermal filler also contains a small amount of non-cross-linked HA, which enhances the delivery of the gel to human tissues and gives the product a low extrusion force (Data on File, RE1210036 Internal Report, Allergan Inc, 2012).

A 15 mg/mL HA dermal filler has been developed using the VYCROSS™ technology platform (developed by Allergan Inc., Irvine, CA, USA) and is formulated using a majority of low molecular weight HA together with a minority of high molecular weight HA (>1 MDa). This formulation has more efficient cross-linking, which affects the rheology of the product in tissues and the hydrophilic properties of the HA gel. The optimized homogenous matrix is smooth rather than granular; this forms a highly malleable gel that is expected to distribute evenly in the treated tissue. These attributes result in a versatile product that can be used not only for lip enhancement, but also to treat fine lines. Finally, the inclusion of mainly low molecular weight HA in the gel, and a lower overall amount of HA, reduces the attraction of water from surrounding tissue, thus reducing the swelling of the gel (Data on File, RE1301025 Internal Report, Allergan Inc, 2012, unpublished data). This HA dermal filler also contains a small amount of non-cross-linked HA, which enhances the delivery of the gel to human tissues and gives the product a low extrusion force (Data on File, RE1210036 Internal Report, Allergan Inc, 2012).

The physiochemical properties of this HA dermal filler allow for effective lip enhancement, with long-lasting duration of up to 12 months. In a prospective, open-label study in 60 subjects by Eccleston *et al*.,^9^ 86.4% and 56.9% of participants treated with the product reported improvement in their lips at 9 and 12 months after treatment, respectively. The product is also associated with high levels of subject satisfaction. In the same prospective study, 94.9%, 93.2% and 89.8% of subjects were reported as satisfied with treatment at 3, 6 and 9 months, respectively.^9^ Subject comfort during treatment has since been improved by the addition of lidocaine which, when formulated with HA gels, has been shown to reduce pain during and after injection and corresponds with increased patient satisfaction.^10^

Despite the high levels of satisfaction with cosmetic lip enhancement, subjects considering whether to undergo this procedure can have a number of concerns, including the potential for swelling, bruising and an unnatural look after treatment. Treatment-emergent adverse events, such as swelling and edema, are considered by the FDA as common side effects of treatment^11^,^12^ and can impact on patients' daily activities, in some cases taking weeks to resolve. In a safety and efficacy study for a common HA filler for lip augmentation, 40% of patients experienced adverse events that affected their daily activity or were disabling, and 15% of patients experienced adverse events (mainly swelling and tenderness) that lasted more than 15 days.^12^

Considering that smoother injections may result in fewer adverse events,^13^ a dermal filler with a smooth consistency and easy delivery to tissues is desirable. With the low swelling ratio, high malleability and low extrusion force of the smooth gel matrix, the HA dermal filler in this study was designed to minimize the risk of swelling and bruising commonly experienced by subjects undergoing cosmetic lip injections,^13^,^14^ and may permit subjects to return to social engagement more quickly. As the long-term efficacy and safety of the product (without Lidocaine) have previously been investigated,^9^ this real-world study aimed to evaluate the short-term aesthetic impact of treatment with this product formulated with Lidocaine. The study was designed to capture outcomes expected to be most relevant to patients and therefore focused on patient-reported outcome assessment. This method allowed subjective evaluation of natural lip enhancement, as opposed to using a numeric lip fullness scale which would only be able to record increases in lip volume. Short-term adverse events that have an effect on the aesthetic outcome, such as bruising and swelling, were also evaluated, including their impact on return to social engagements.

## Materials and methods

### Study design

This one-month, prospective, open-label, multicenter, observational postmarket study (clinicaltrials.gov identifier: NCT01629134) was conducted in two German sites. Eligible subjects were aged 18 years and older, expressed a desire and willingness for correction of asymmetry or enhancement of their lips and could comply with the study requirements. Exclusion criteria comprised incompatibility with the prescribing criteria for the product and the presence of a condition or a situation deemed unsafe for the subject or unfavorable to the subject's participation in the study (e.g. untreated epilepsy, tendency to develop hypertrophic scarring, porphyria and hypersensitivity to HA, lidocaine or amide-type local anesthetics; the full list can be found in the product's directions for use [DFU]). Each subject signed an informed consent form and underwent treatment with the product.

### Study protocol

Each subject attended two visits to the investigation site. At the first visit (Day 0), the subjects underwent a pretreatment evaluation by the physician to determine the appropriate injection procedure and volume based on clinical experience, with the aim of achieving optimum correction. Demographic information was collected for each subject. Subsequently, subjects received Juvéderm® VOLBELLA® with Lidocaine injections in line with the product's DFU.

To restore contour and definition to the lip, horizontal linear injections were performed at the vermilion border. A retrograde technique with a 30G needle using up to three injection points on each side of the lip (upper and lower) was used. The corner of the mouth was treated using a bolus of product with one injection site, which was massaged for optimal placement.

To restore volume to the body of the lip, injections were performed transcutaneously (either through the white lip or through the red lip just inferior to the vermillion border). A 30G needle (using up to three injection points on each side of the lip) was used to inject on average 0.05–0.25 mL of product in a bolus into the body of the lip. This was then massaged for optimal placement.

At the follow-up visit, within 4 weeks following the initial treatment, an optional top-up injection could be performed if the physician judged that optimal correction had not been achieved, or if the subject requested it and a retreatment was considered clinically indicated by the injector.

**Figure 1:**
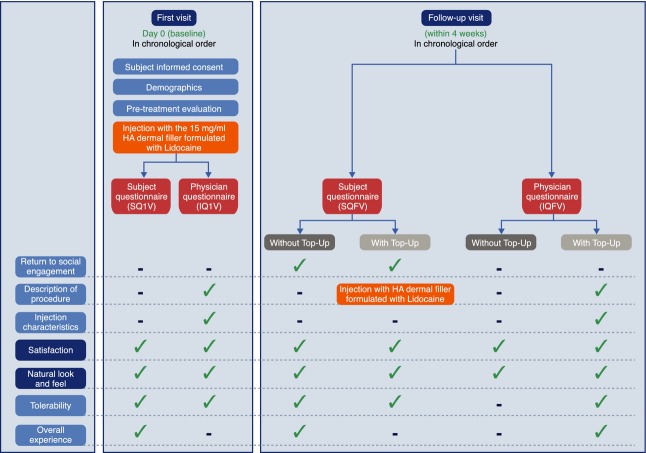
Treatment protocol and evaluations. Primary endpoints are dark blue; secondary endpoints are light blue. HA: Hyaluronic acid; IQ1V: Injector Questionnaire – First Visit; IQEOS: Injector Questionnaire – End of Study; IQFV: Injector Questionnaire – Follow-up Visit; SQ1V: Subject Questionnaire – First Visit; SQFV: Subject Questionnaire – Follow-up Visit.

**Figure 2:**
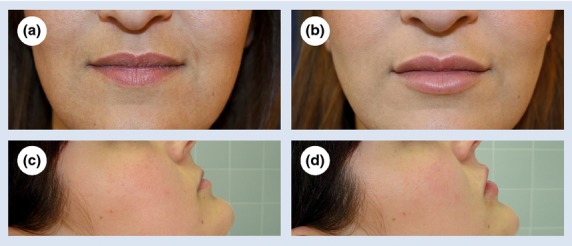
Lips of patient 1 (a) before treatment and (b) post-treatment with the HA dermal filler at the follow-up visit. Lips of patient 2 (c) before treatment and (d) post-treatment with the HA dermal filler at the follow-up visit.

At each visit, the subjects and their physicians completed questionnaires before and after injection to assess the aesthetic impact of treatment, other characteristics related to the injection(s) and safety endpoints (Fig. 1). Assessment of bruising and swelling was performed 10–15 min after administration of the injection at the first visit, and retrospectively over the preceding 4 weeks at follow-up visit. Photographs of the subject's face were taken before and/or after injection if deemed appropriate by the physician (Fig. 2).

Subjects completed a questionnaire on their overall experience of the product at each visit, and physicians completed a questionnaire at the second visit only.

### Study subpopulations

Some, but not all, subjects underwent a top-up injection at follow-up; therefore, different questions were applicable to these subpopulations. Most of the analyses on data collected at the follow-up visit were also performed separately for subjects with and without top-up treatment.

### Endpoints and statistical analyses

#### Primary endpoints

The primary study objective was to evaluate the short-term aesthetic impact of treatment with the HA dermal filler when formulated with Lidocaine for enhancement of the lips or correction of asymmetry of the lips. The primary endpoints defined were patient satisfaction with improvement in lips and satisfaction with the natural look and feel of lips immediately after injection and at the follow-up visit within 4 weeks post-treatment.

The level of satisfaction with improvement in the lips was evaluated at the first visit and at the follow-up visit using a 6-point scale from *Extremely Satisfied* to *Very Dissatisfied*.

The rating of the natural look and feel of the lips was evaluated at the first visit and at the follow-up visit using a four-point scale from *Extremely Natural* to *Not Natural*.

#### Secondary endpoints

Secondary endpoints were defined to provide a complete overview of subject and physician experience, and included tolerability (as assessed by comfort of injection, bruising and swelling), injection characteristics (ease of injection, need for massage and malleability), time taken to return to social engagements, comparative evaluation with previous treatments, overall experience and safety (Table 1).

**Table 1:** Clinical investigation endpoints: secondary endpoints

Secondary endpoints	
Tolerability	
Injection discomfort	
Subject experience	11-point scale from 0 = *No Discomfort* to 10 = *Extreme Discomfort*
Physician experience	11-point scale from 0 = *No Discomfort* to 10 = *Extreme Discomfort*
Bruising in lips	
Subject experience	5-point scale from *None* to *Considerable*
Physician experience	5-point scale from *None* to *Considerable*
Swelling in lips	
Subject experience	7-point scale from *No Swelling* to *Swelling Not Resolved Yet* (subjects without top-up treatment only)
Physician experience	7-point scale from *No Swelling* to *Swelling Not Resolved Yet*
Injection characteristics	
Ease of use	
Physician experience	11-point scale from 0 = *Very Easy* to 10 = *Extremely Difficult*
Malleability	
Physician experience	11-point scale from 0 = *Extremely Malleable* to 10 = *Not Malleable*
Need for massage	
Physician experience	4-point scale from *None or Minimal* to *A Lot*
Return to social engagement	
Subject experience	5-point scale from <1 day to >6 days
Overall experience	
Physician experience	4 questions
Subject experience	2 questions (additional 2 questions for subjects without top-up treatment only)
Safety	
Subject experience	Evaluated by subject-reported adverse events and serious adverse events, directly related to the device, which occurred during the study and/or the follow-up period

IQ1V: Injector Questionnaire – First Visit; IQEOS: Injector Questionnaire – End Of Study; IQFV: Injector Questionnaire – Follow-up Visit; SQ1V: Subject Questionnaire – First Visit; SQFV: Subject Questionnaire – Follow-up Visit.

### Statistical analyses

Analyses were primarily descriptive. Quantitative variables were described by their total (*n*), number of missing data (answers to questionnaire points not available), mean, standard deviation, median, quartiles 1 and 3 and extreme values. Categorical variables were described by the absolute and relative (%) frequency of each class and number of missing data. Missing data were not taken into account in the calculation of percentages.

## Results

### Demographics

The study population included 62 subjects (30 from Center 1 and 32 from Center 2) who had been selected by the site physician, signed an informed consent form and underwent treatment with the HA dermal filler. In total, 62 physician questionnaires and 62 subject questionnaires were collected at the first visit, with 61 physician and 61 subject questionnaires at the follow-up visit. Both physicians in this study completed the End of Study Questionnaire. The mean age of the study population was 39.7 years (range: 21–75 years) and 55 of the total 62 subjects were female (88.7%). When surveyed before beginning treatment with the product, 77.4% of subjects expressed concerns regarding a possible unnatural look after treatment and 19.4% had concerns regarding possible discomfort of injection. Other concerns (possible swelling, bruising, etc.) were reported by 27.4% of subjects.

Of the 62 subjects included in the study, 17 subjects (27.9%) were treated with a top-up injection at the follow-up visit.

At first visit, the total volume of product injected was on average 1.39 mL (min: 1.0 mL, max: 3.0 mL). At follow-up visit, for subjects who opted for top-up treatment, the total volume injected was on average 0.41 mL (min: 0.1 mL, max: 1.0 mL).

### Primary endpoints

#### Satisfaction with improvement in lips

##### Subject satisfaction

**Figure 3:**
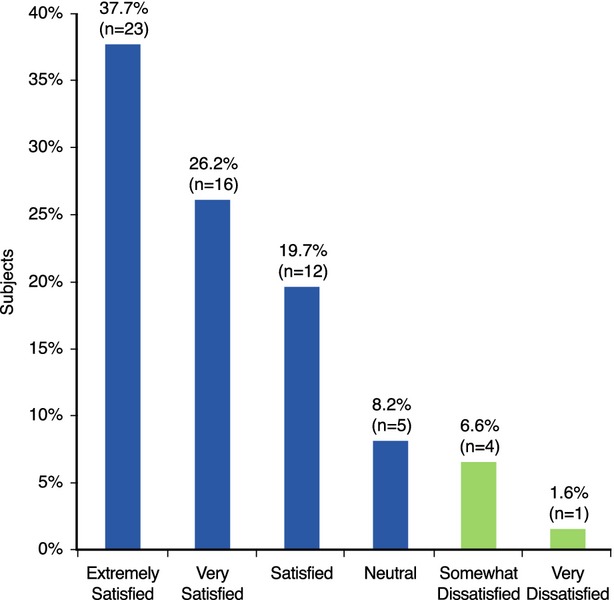
Subject satisfaction with improvement in lips following treatment with the HA dermal filler at first visit.

For the primary endpoint, 83.6% of subjects (*n* = 51 out of 61) were at the first visit *Extremely Satisfied*, *Very Satisfied* or *Satisfied* with the improvement in their lips following treatment with the HA dermal filler (Fig. 3 and Table 2a).

**Table 2:** (a) Satisfaction with improvement in lips (b) Rating of the natural look and feel of the lips) at first visit and follow-up

	Primary endpoint		
	
	First visit (*n* = 62) (%)*	Follow-up visit without top-up (*n* = 44) (%)†	Follow-up visit with top-up (*n* = 17) (%)‡
(a) Satisfaction with improvement in lips			
Subject experience			
Missing data§	1	1	0
Extremely satisfied	23 (37.7)	21 (48.8)	8 (47.1)
Very satisfied	16 (26.2)	16 (37.2)	7 (41.2)
Satisfied	12 (19.7)	3 (7.0)	1 (5.9)
Neutral	5 (8.2)	2 (4.7)	1 (5.9)
Somewhat dissatisfied	4 (6.6)	1 (2.3)	0 (0.0)
Very dissatisfied	1 (1.6)	0 (0.0)	0 (0.0)
Physician experience			
Extremely satisfied	17 (27.4)	23 (52.3)	1 (5.9)
Very satisfied	45 (72.6)	21 (47.7)	16 (94.1)
Satisfied	0 (0.0)	0 (0.0)	0 (0.0)
Neutral	0 (0.0)	0 (0.0)	0 (0.0)
Somewhat dissatisfied	0 (0.0)	0 (0.0)	0 (0.0)
Very dissatisfied	0 (0.0)	0 (0.0)	0 (0.0)
(b) Rating of the natural look and feel of the lips			
Subject experience			
Missing data§	2	1	1
Extremely natural	9 (15.0)	24 (55.8)	7 (43.8)
Very natural	28 (46.7)	16 (37.2)	5 (31.3)
Slightly natural	16 (26.7)	3 (7.0)	4 (25.0)
Not natural	7 (11.7)	0 (0.0)	0 (0.0)
Physician experience			
Extremely natural	19 (30.6)	24 (54.5)	4 (23.5)
Very natural	43 (69.4)	20 (45.5)	13 (76.5)
Slightly natural	0 (0.0)	0 (0.0)	0 (0.0)
Not natural	0 (0.0)	0 (0.0)	0 (0.0)

* Evaluation just after treatment.

† Evaluation 4 weeks post-treatment.

‡ Evaluation just after top-up treatment.

§ Answers to these questionnaire points not available.

In the pretreatment evaluation, 28 subjects were *Somewhat Unhappy* or *Extremely Unhappy* with their lips. After treatment, 25 (89.3%) of these subjects were *Extremely Satisfied*, *Very Satisfied* or *Satisfied* with improvement in their lips, and the remaining 10.7% reported *Neutral* satisfaction.

At follow-up visit, 93.0% of subjects (*n* = 40 out of 43) who did not opt for top-up injection were *Extremely Satisfied*, *Very Satisfied* or *Satisfied* with improvement in their lips. Satisfaction of subjects without top-up remained stable between the first visit and the follow-up visit in 45.2% of subjects and was improved in 33.3% of subjects.

Of 94.1% (*n* = 16 out of 17) of subjects who opted for top-up treatment were *Extremely Satisfied*, *Very Satisfied* or *Satisfied* with improvement in lips immediately after top-up injection at the follow-up visit. Satisfaction of subjects with top-up remained stable between the first visit and the follow-up visit in 47.1% of subjects, and was improved in 41.2% of subjects.

##### Injector satisfaction

Both physicians were *Extremely Satisfied* or *Very Satisfied* with the improvement in their subjects' lips following the first treatment, as well as at follow-up visit in subjects' lips with and without top-up (Table 2a).

#### Rating the natural look and feel of the lips

##### Subject satisfaction

**Figure 4:**
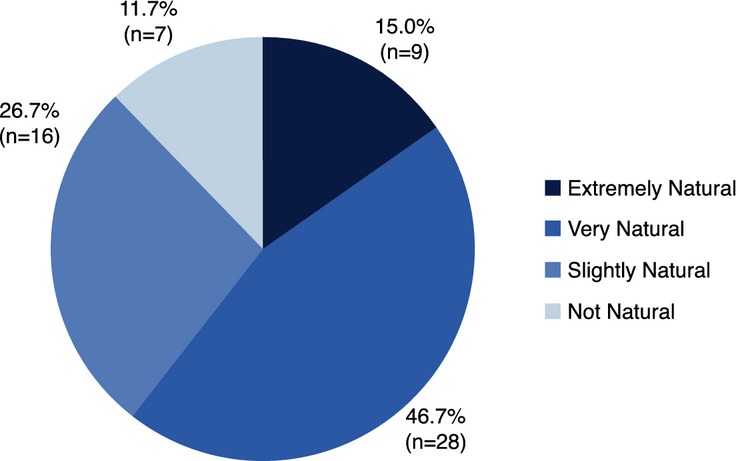
Subject rating of natural look and feel of lips following treatment with the HA dermal filler at first visit.

When rating the look and feel of the lips after the first injection, 61.7% of subjects (*n* = 37 of 60 subjects available for follow-up) considered the treatment looked and felt *Extremely Natural* (15%) or *Very Natural* (46.7%) (Fig. 4, Table 2b).

Among subjects without top-up injection, 65.1% of subjects (*n* = 28 out of 43) rated the look and feel of their lips as *Extremely Natural* or *Very Natural* just after treatment at first visit, and this increased to 93.0% at the follow-up visit. Comparably, in subjects who received top-up treatment, the look and feel of the lips was reported as *Extremely Natural* or *Very Natural* by 50.0% of subjects (*n* = 8 out of 16) just after treatment at first visit, which increased to 75.0% at follow-up visit.

No subject in the study rated the look and feel of their lips as *Not Natural* at the follow-up visit.

##### Injector satisfaction

Physicians rated the look of their subjects' lips following treatment with the product as *Extremely Natural* or *Very Natural* (Table 2b).

### Secondary endpoints

#### Bruising

At the first visit, 95% of subjects (*n* = 57 out of 60) reported *Little* or *No* bruising. Comparable results were also reported by the physicians, with *Little* or *No* bruising reported in 98.4% of cases (*n* = 61 of 62). At follow-up, 47.7% of subjects who did not receive top-up treatment (*n* = 21 of 44) and 66.7% of subjects with top-up treatment (*n* = 10 of 15) reported *No Bruising* since the first visit. Of the remaining subjects who experienced some level of bruising (52.3% in the without top-up treatment group; 33.3% in the top-up treatment group), the bruising resolved in all cases within 5–6 days.

#### Swelling

**Figure 5:**
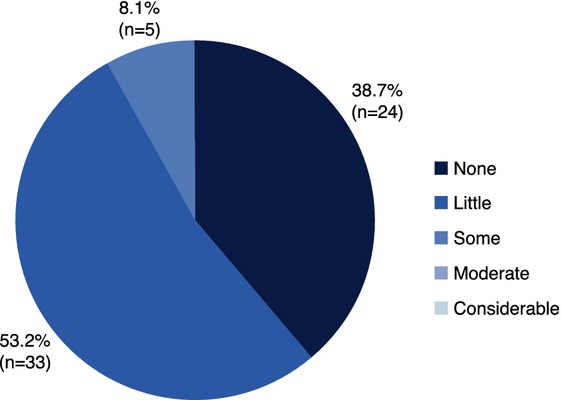
Physician rating of swelling in subjects' lips just after treatment with the HA dermal filler at first visit.

Physicians reported that in 91.9% of cases (*n* = 57 out of 62), subjects experienced *No Swelling* (38.7%) or *Little Swelling* (53.2%) of their lips after the first injection; all swelling resolved within 5–6 days (Fig. 5). Among subjects who did not receive a top-up injection (*n* = 44), swelling resolved within 1–2 days in 79.6% of cases. Among subjects receiving top-up injection at the follow-up visit, physicians reported that 100% of subjects (*n* = 17) experienced *No Swelling* (35.3%) or *Little Swelling* (64.7%) in the lips after top-up treatment.

#### Return to social engagement

**Figure 6:**
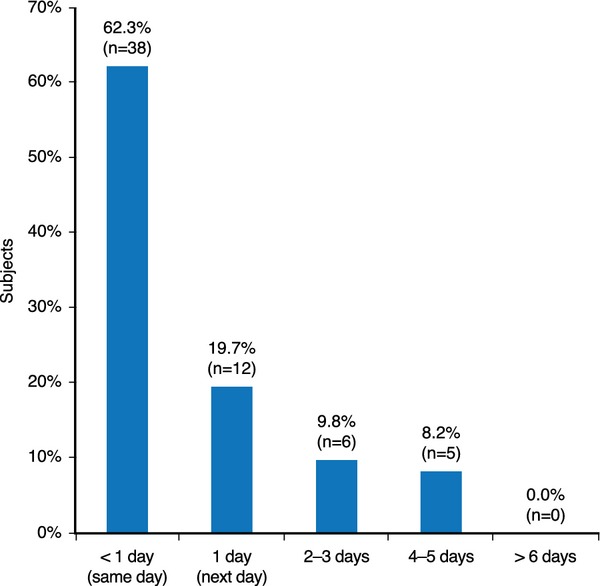
Subjects' return to social engagement after treatment with the HA dermal filler at first visit.

After receiving treatment with the product, 62.3% of subjects returned to social engagement on the same day as the procedure. All subjects returned to social engagement within 4–5 days (Fig. 6).

#### Comfort of injection

Subjects and physicians considered administration of the product to cause little discomfort during the first injection. On an 11-point scale, where 0 = no discomfort and 10 = extreme discomfort, both subjects and physicians reported similar mean levels of discomfort during the first injection (1.3 [± 1.5] and 1.2 [± 1.0], respectively). No or mild discomfort (level of discomfort ≤2) was reported by 78.3% of subjects and in 95.2% of cases by the physicians. Additional anesthesia was given to 22 subjects in the study (35.5%); regional anesthesia (mepivacaine hydrochloride 5 mL) was used in 14 subjects, and topical anesthesia (lidocaine cream) was used in 8 subjects.

#### Injection characteristics

**Figure 7:**
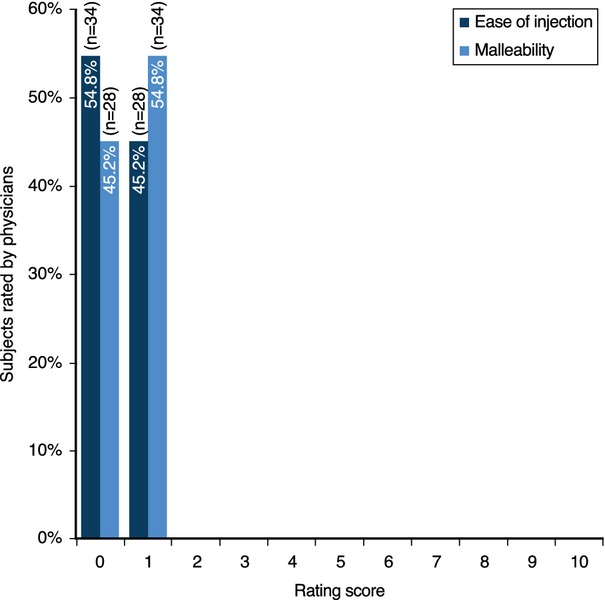
Physician rating of ease of injection* and malleability*^*^ when using the HA dermal filler. *Ease of injection rated on a scale from 0 (*Very Easy*) to 10 (*Extremely Difficult*). **Malleability rated on a scale from 0 (*Extremely Malleable/Not Hard to Mold*) to 10 (*Not Malleable/Hard to Mold*).

**Figure 8:**
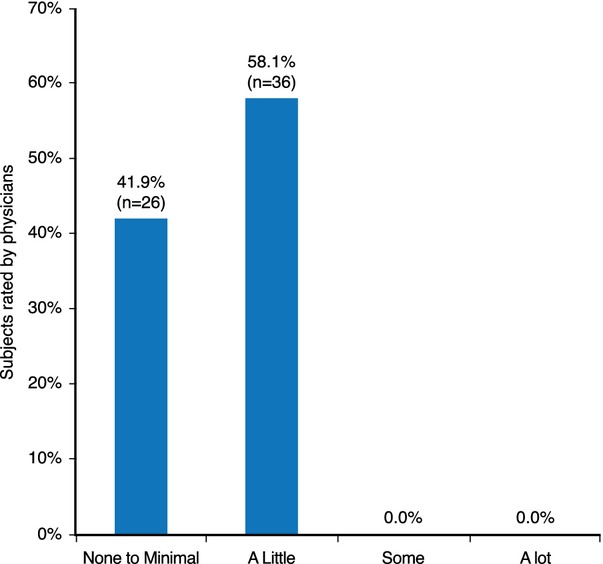
Physician rating of need for massage with the HA dermal filler.

Based on the injection at the first visit, physicians considered the product to be very easy to inject, easy to mold/sculpt (Fig. 7), and to require little or no massage (Fig. 8) when asked to rate using rating scales (ease of injection rated on a scale from 0 [*Very Easy*] to 10 [*Extremely Difficult*], and malleability rated on a scale from 0 [*Extremely Malleable/Not Hard to Mold*] to 10 [*Not Malleable/Hard to Mold*]).

#### Overall experience

After injection at first visit, 96.7% of subjects stated that they would use this HA dermal filler treatment again in the future, and 100% of the subjects and physicians in the study stated they would recommend the treatment to others.

#### Safety

Aside from procedure-related adverse events (i.e. low reported bruising and swelling), no other adverse events were reported during the study.

## Discussion

Overall, the HA dermal filler in this study was very well accepted as a new treatment option for the lips by both subjects and physicians, with 83.6% of the subjects being *Extremely Satisfied*, *Very Satisfied* or *Satisfied* with the improvement in their lips after the first injection, and the physicians being *Extremely Satisfied* or *Very Satisfied* with lip improvement in 100% of the subjects. The high levels of satisfaction may be explained by the natural effect produced by the treatment: 61.7% of subjects rated the look and feel of their lips as *Extremely Natural* or *Very Natural* after the first injection, and no subject considered the look and feel to be *Not Natural* at the follow-up visit. These results are particularly relevant when it is considered that the majority of subjects (77.4%) had pretreatment concerns about the possible unnatural look of their lips following injection.

The natural look and feel of the lips after treatment may be linked to the physiochemical properties of the product. Unlike gel particle formulations that have a granular consistency, the VYCROSS™ platform produces a smooth dermal filler that is highly malleable and easy to spread in the lips. The smooth consistency of the gel, combined with the inclusion of a small amount of non-cross-linked HA in the product's formulation, may also explain why the physicians in this study considered the product to be very easy to inject and to mold/sculpt and to require little or no massage.

Subjects undergoing lip enhancement procedures can expect treatment-emergent adverse events, such as swelling and bruising. These can affect daily activities and lengthen the time taken to return to social engagements while waiting for these side effects to resolve.^12^ As such, low levels of swelling and bruising are important to ensure subject satisfaction with the aesthetic lip enhancement and rapid return to social engagements. In this study, treatment was well tolerated, with subjects reporting little discomfort during the first injection, and with low rates of swelling and bruising after treatment. These results may be related to the rheological and hydrophilic properties of the product. It has also been observed that adverse events experienced by patients undergoing lip enhancement can be linked to factors relating to injection technique, such as the rapid injection of a large volume of filler.^13^ It was noted during this study that the product was easy to inject, which may have also contributed to the low incidence of these adverse events, such as swelling and bruising. Following treatment, physicians reported that subjects experienced *No Swelling* or *Little Swelling* of their lips after the first injection in 91.9% of cases. Almost two-thirds (62.3%) of subjects were able to return to normal activities on the same day, with a further 19.7% able to return within 1 day.

There were several limitations of this descriptive study. Lack of a control group and blinding make correlations between the physical properties and clinical outcomes speculative, but common to many studies evaluating filler treatments, the use of placebo or blinding is often not practical. Potential confounding variables may include subtle variations in injection technique. As previously mentioned, additional limitations include the use of nonvalidated scales and lack of precise outcome definitions (e.g. swelling). However, because the aim of the study was to evaluate lip enhancement using the product in a real-world setting, this subjective patient-centric outcome evaluation was considered to be the most suitable approach, particularly as no objective scale exists for assessment of the natural look of lip enhancement. Lip fullness scales were not considered to be appropriate for the study design. The relatively limited follow-up period may be considered a limitation of the study; however, because the long-term efficacy and safety of the product (without Lidocaine) have previously been investigated,^9^ this one-month study was designed to capture any immediate adverse events, their effect on return to social engagement and the short-term efficacy of the product, thereby adding to the body of clinical experience with this HA dermal filler.

## Conclusion

Lip enhancement with this 15 mg/mL HA dermal filler formulated with Lidocaine was associated with very high levels of subject satisfaction and was considered to result in a natural look and feel of the lips. The treatment was well tolerated, and the levels of bruising and swelling were low enough to allow the majority of subjects to return to social engagements on the same day as treatment.
